# Ethnicity and breast cancer incidence in over 329,500 women in England in 2011-2019

**DOI:** 10.1016/j.ejso.2025.109585

**Published:** 2026-02

**Authors:** T Gathani, SW Kan, S Sweetland, G Reeves

**Affiliations:** 1 Cancer Epidemiology Unit, Nuffield Department of Population Health, University of Oxford; 2 Oxford University Hospitals NHS Foundation Trust

**Keywords:** Breast cancer, incidence, ethnicity, England, Census

## Abstract

**Introduction:**

Previous studies have reported an overall lower breast cancer incidence in women from Asian and Black backgrounds compared with white women. Age standardised and age specific incidence rates in the largest specific ethnicities within Asian and Black groups are not reported.

**Materials and methods:**

Data on population size and the age distribution of women in five ethnic groups of interest (white British, Black African, Black Caribbean, Indian and Pakistani) were extracted from the Office for National Statistics 2001, 2011 and 2021 census data for England.

Cancer registrations for invasive breast cancer (ICD-10 C50) in women in England aged ≥25 years during 2011-2019 with a recorded ethnicity were extracted from the National Cancer Registration and Analysis Service.

Age standardised (ASIRs) and age specific (ASRs) incidence rates in five ethnic groups of interest were calculated.

**Results:**

329,655 women who were aged ≥25 years and in one of the five ethnic groups of interest had a record of an incident C50 cancer registration during 2011-2019. The ASIR was highest for white women (199.6 (95% CI 198.9-200.3)), and lowest for Black African women (118.2 (95% CI 111.6-125.1)). The ASRs for invasive breast cancer were generally lower in women from minority ethnic groups compared to white women in all age groups examined except for younger Black Caribbean women.

**Conclusions:**

There are significant differences in breast cancer incidence rates between women from specific ethnicities. This requires further investigation in large scale prospective studies considering potential differences by ethnicity in known risk factors for breast cancer.

## Background

Breast cancer is the most commonly diagnosed cancer in England in women of all ethnicities [1]. Approximately 45,000 women are registered with incident invasive breast cancer (ICD-10 C50) in England annually [2].

The Office for National Statistics (ONS) conducts a census every 10 years to provide information about households in England and Wales, and includes information about ethnicity [3]. Ethnicity was first recorded in the 1991 census, and the coding system has evolved over time [4, 5]. Ethnicity is recorded in five aggregate groups including Asian, Black, Mixed, White and Other. Within these five aggregate groups, there are 19 standardised ethnic groups (Table 1). Since the 2001 census, Black African, Black Caribbean, Indian and Pakistani have been consistently recorded as distinct ethnic groups. In the 1991 census 94% of respondents identified as white British compared to 82% in 2021.

Although previous studies have reported a lower overall breast cancer incidence in women from Asian and Black backgrounds [1, 6], the age standardised and age specific incidence rates in the ethnicities within these aggregate groups i.e. Indian and Pakistani, Black African and Black Caribbean, are not reported. Previous studies are also limited by a lack of reliable population data by ethnic group [1], but the availability of the 2021 census data by ethnicity allows for more robust analysis to be conducted as current rather than projected population estimates are available.

Women are largely diagnosed with breast cancer either because of presenting with symptoms such as a breast lump, or through population based mammographic screening depending on their age. Women of any age can be referred to secondary care services for assessment of breast symptoms [7]. Routine three yearly mammographic screening is offered to women aged 50-70 years who are registered with a general practitioner [8]. Roughly six out of ten women are diagnosed with breast cancer as a result of symptoms, and one in three women are diagnosed following screening [9]. However, among women of screening age, roughly 60% of breast cancer diagnoses follow routine screening [10]. Variations by ethnicity in the frequency of presentation with symptoms or in the uptake of screening invitations could have a role in explaining any observed differences in breast cancer incidence in these groups.

There is increasing interest in addressing health inequalities due to ethnicity in the National Health Service in England [11]. Understanding variations in breast cancer incidence by ethnicity and by age can help inform the planning and delivery of breast cancer services to address any apparent inequalities.

In this paper, we present census data, age standardised and age specific breast cancer incidence rates for women from the five largest ethnic groups, (Black African, Black Caribbean, Indian, Pakistani and white British) in England.

## Methods

### Data sources

All data used in this analysis are extracted from either the Office for National Statistics (ONS) for census data or from the National Cancer Registration and Analysis Service (NCRAS) for information on cancer incidence.

Data on the age distributions for women recorded as Black African, Black Caribbean, Indian, Pakistani or white British were extracted from the ONS 2001 [12], 2011 [13] and 2021 [14] census data for England. For the 2001 census data, age distributions in the different ethnic groups are only available in 5 year age bands. For the 2011 and 2021 censuses a bespoke dataset of age in single years and ethnic groups was provided on request to the Census Customer Services team at the Office of National Statistics and used to estimate the median age of the population in each ethnic group.

All cancer registrations for invasive breast cancer (ICD-10 50) in women in England diagnosed between 1 January 2011 and 31 December 2019 were extracted from NCRAS. A detailed description of the data resource profile is provided elsewhere [15, 16]. Where ethnicity was recorded, women were assigned to one of the five largest ethnic groups in the 2011 and 2021 census [17]: Black African, Black Caribbean, Indian, Pakistani or white.

### Statistical Analysis

Using the 2011 and 2021 census data, an average annual population estimate was estimated for women aged ≥25 years for each of the five ethnic groups of interest.

The average annual number of women registered with incident C50 was estimated for Black African, Black Caribbean, Indian, Pakistani and white British women during 2011-2019 in England. If a woman had more than one registration of C50 over the time period of interest, only the first recorded incidence contributed to the analyses.

Age standardised breast cancer incidence rates (ASIRs) per 100,000 women and their 95% confidence intervals for each of the five ethnic groups of interest were estimated using direct standardisation to the European Standard Population [18]. Differences in the ASIRs between two ethnic groups were tested using a chi-squared heterogeneity test.

Age-specific rates (ASRs) and their 95% confidence intervals were estimated using Poisson regression for each of the five ethnic groups. This was done in three defined age groups which relate to the potential route to a breast cancer diagnosis: women aged 25-49 and aged 70+, who will be diagnosed largely as a result of symptoms; and women aged 50-69 years, who could be diagnosed as a result of symptoms or following routine invitation for mammographic screening. Differences in the ASRs between two ethnic groups were tested using a Wald test.

All analyses were conducted in Stata Version 18.5.

## Results

The number of women and their median age in each of the five ethnic groups in the 2001, 2011 and 2021 census in England is shown in Table 2. Between the 2001 and 2021 census, there has been a large increase in the number of Black African, Indian and Pakistani women, whilst the number of Black Caribbean women has remained largely similar. The number of Black African women has tripled from ~250,000 in 2001 to over 750,000 in 2021. The total number of Indian and Pakistani women has almost doubled over the same time period.

The median age of women has increased over the three censuses in all ethnic groups. White women, on average, are generally older compared to all the other ethnic minority groups except women from Black Caribbean backgrounds. In 2021, white women had a median age of 44 years compared to 36 years in Indian women, 31 years in Black African women, and 28 years in women from Pakistani backgrounds. Black Caribbean women are, on average, the oldest of the ethnic minority groups with a median age of 32 years in 2001, which rose to 45 years in 2021.

Figure 2 shows the age distribution in four broad categories of women in the five ethnic groups in the three censuses.

In 2021, over 80% of Black African and Pakistani women were aged under 50 years of age, compared to less than 60% of white and Black Caribbean women, and just over 70% of Indian women. Only 16% of Black African and 12% of Pakistani women were within the age range to be invited for routine mammographic screening (50-69 years), compared to a fifth of Indian women, a quarter of white women and a third of Black Caribbean women. The proportion of older women (70+ years) was 17% for white women compared to only 2% of Black African women.

Of note, among Black African and Black Caribbean women the proportion of women eligible for population-based screening roughly doubled between 2001 and 2021 (8% versus 16% for Black African women, and 18% versus 33% for Black Caribbean women). In comparison, the proportion of Indian and Pakistani women eligible for screening only rose by 3% for both groups (16% versus 19% for Indian women, and 9% versus 12% for Pakistani women). The proportion of older white women remained relatively constant over the three censuses with 15% aged 70+ years in 2001 compared to 17% in 2021, whereas the proportion of Black Caribbean women aged 70+ years doubled in the same time period, from 5% in 2001 to 12% in 2021.

The average number of incident cases per year and the age standardised incidence rates (ASIRs) for C50 breast cancer by ethnic group are shown in Table 3. During 2011-2019 in England among women aged ≥25 years, there were an average of 35 127 incident breast cancer cases per year among white women, 282 cases among Black African women, 346 cases among Black Caribbean women, 569 cases among Indian women and 304 cases among Pakistani women.

Overall, white women had a higher breast cancer incidence rate (ASIR 199.6 (95% CI 198.9-200.3)) compared to women from ethnic minority backgrounds. Indian and Pakistani women had similar incidence rates for breast cancer (ASIR 134.7 and 137.3 respectively). However, significant differences (p_heterogeneity_<0.001) were observed in the incidence rates of breast cancer for Black Caribbean women (ASIR 146.0 (95% CI 140.7-151.3)) compared to Black African women (ASIR 118.2 (95% CI 111.6-125.1)).

Age specific rates (ASRs) for C50 breast cancer are shown in Figure 2. For women from all ethnic groups, as expected, there was an observed increased incidence of breast cancer with increasing age. In each age range of interest, white women had a higher ASR compared to women from the minority ethnic groups, except for younger white and Black Caribbean women (ASR 83.0 (95%CI 82.3-83.7) versus 79.4 (95%CI 74.3-84.9) respectively among those aged 25-49 years; p_heterogeneity_=0.2).

Indian and Pakistani women had similar ASRs within each of the age groups examined. However, in comparison, differences in ASRs were observed between Black African and Black Caribbean women who were younger (ASR 51.9 (95% CI 49.2-54.8) versus ASR 74.3 (95% CI 74.3-84.9) respectively; p_heterogeneity_<0.001), or of screening age (ASR 129.3 (95% CI (121.5-137.6) versus ASR 181.6 (95% CI 172.5-191.3) respectively; p_heterogeneity_<0.001). However, the ASRs were similar for Black African and Black Caribbean women among those aged 70+ years.

## Discussion

In this large study using national census and cancer registration data, there were clear differences observed in the population demographics for women from Indian and Pakistani, and Black African and Black Caribbean backgrounds compared to white women. Furthermore, women from these ethnic groups, except for younger Black Caribbean women, had a lower incidence of breast cancer compared to white women, both overall and in the separate age groups examined.

Among the South Asian ethnic groups, the incidence rates of breast cancer in Indian and Pakistani women were similar in all age groups examined. Among the Black ethnic groups, breast cancer ASRs were most similar in the older age group, but the ASRs for Black African women were significantly lower in the younger age group and in women of screening age compared to Black Caribbean women. To our knowledge, these findings have not been previously described and provide valuable additional information about variations in breast cancer incidence in ethnic groups.

Understanding the history of migration patterns into the United Kingdom helps to explain the observed differences in the age distributions of women from different ethnic backgrounds and informs what future distributions may look like.

There is a long history of migration into the UK from both Black and South Asian groups, but prior to 1948 the overall numbers were relatively small. In the 1951 census only 4% of the population reported being born in a non-UK country, compared to 16% in 2021 [19]. Migration was actively encouraged following the end of the Second World War driven by the need to rebuild the country. The 1948 British Nationality Act made way for large numbers of people to migrate from the colonies of the British Empire, by granting these citizens the right to live and work in the UK without restriction [20]. Examination of legislation that follows shows that the various acts later introduced not only restricted the number of migrants that entered the UK, but also began to protect them through the introduction of anti-discrimination laws.

The patterns of migration vary among the ethnic groups of interest. Among Black populations, Black Caribbean migration largely occurred in the 1950s and 1960s, in comparison to Black African migration which became increasingly significantly from the 1980s onwards [21, 22]. Most of the early migration from the Caribbean was to work in the newly formed National Health Service, and other national institutions such as the post office and public transport, whereas the purpose of Black African migration was largely for education [21, 22]. Black African migration due to political instability in various African countries has increased in the recent past, but these numbers as a proportion of the total number of migrants are low [23].

Similarly, although there is a long history of South Asians living in Britain prior to 1948 these numbers were small. Large-scale migration from South Asia to Britain began in the 1950s and peaked in the late 1960s and early 1970s as immigration polices became more restrictive but did continue thereafter. Examination of data collected on country of birth in the 2011 and 2021 censuses shows that India and Pakistan are two of the most common non-UK countries of birth for usual residents in the UK [19].

Until 2019 European Union (EU) nationals were a very large component of total long-term migration into the UK. However since 2021 when free movement ended for EU nationals, the majority of immigration now comprises non-EU nationals [24]. Since 2019, the number of Indian, Nigerian and Pakistani nationals arriving in the UK has seen the largest increase. There were approximately 62,000 more Pakistani nationals, 127,000 more Nigerian nationals and 178,000 more Indian nationals immigrating to the UK in 2023 compared with 2019. These migrants are a mixture of skilled workers, a large proportion of whom are employed in the health and social care sectors, and students in the UK on study-related visas [25].

**Figure 1:**
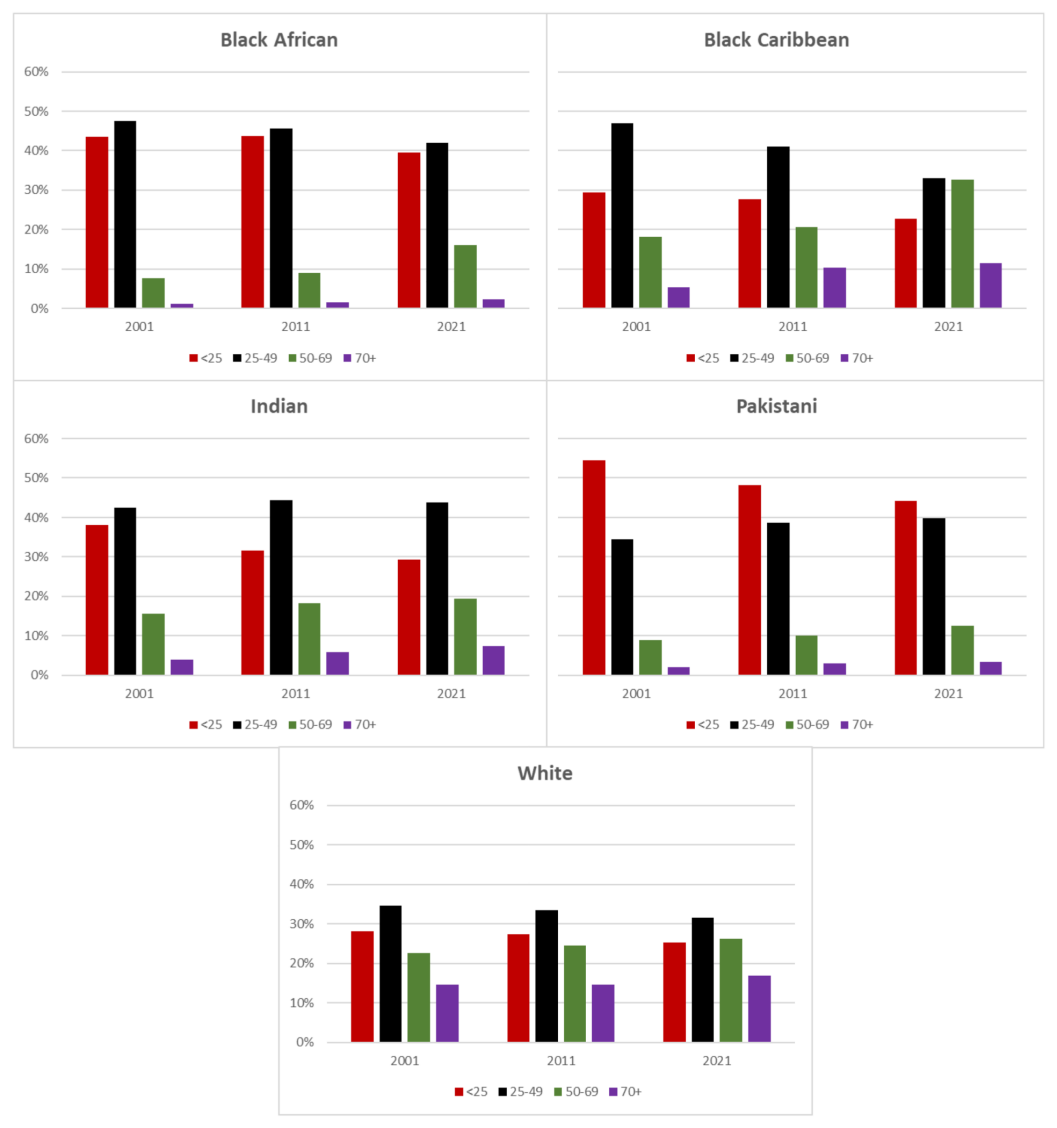
The age distribution of Black African, Black Caribbean, Indian, Pakistani and white women in the 2001, 2011 and 2021 national census

**Figure 2:**
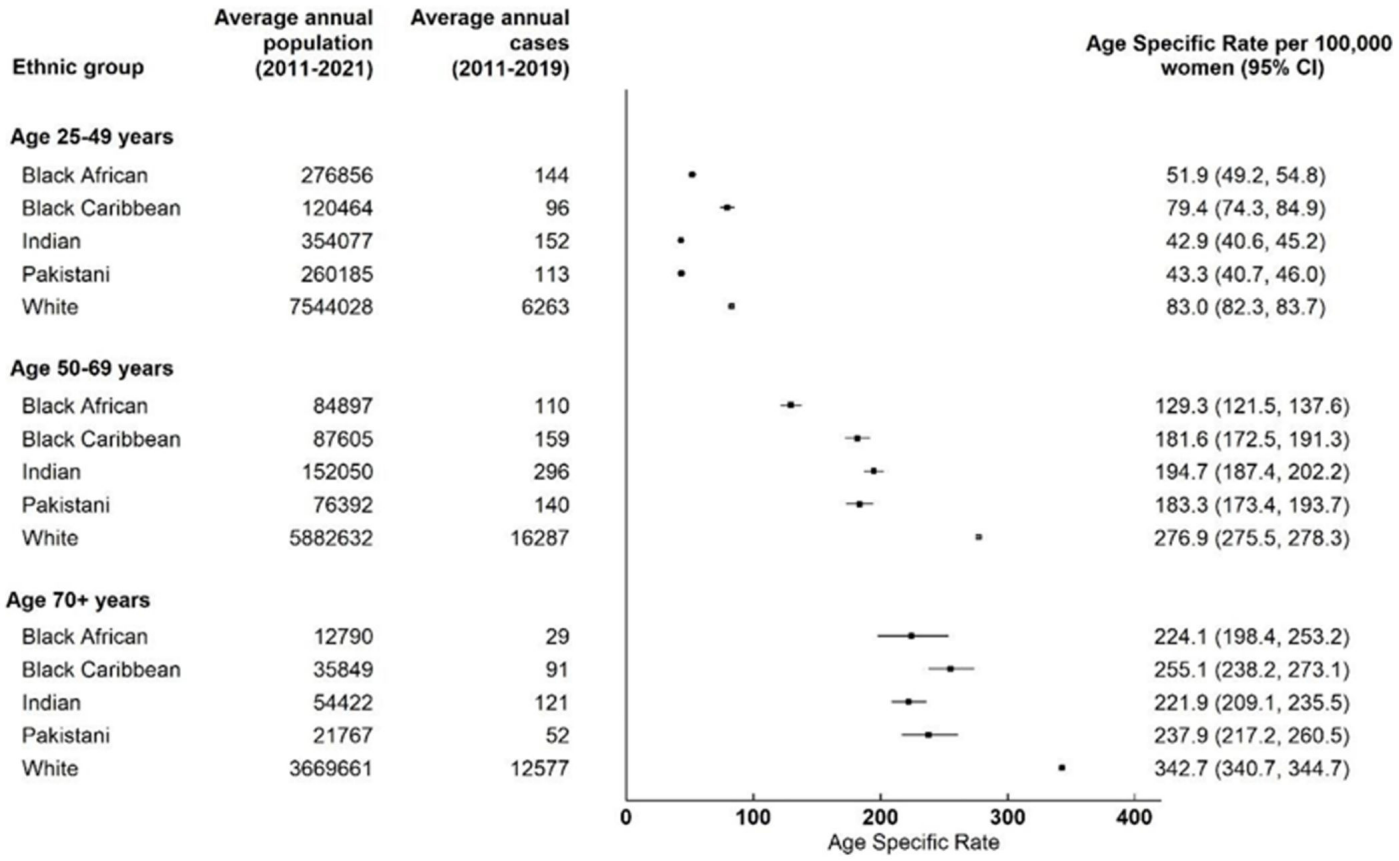
Age specific rates for C50 breast cancer by ethnicity in women aged ≥25 years in England during 2011-2019.

As migrants generally tend to be young, the average age of populations from ethnic groups will be younger compared to white people, as shown in Figure 1 and Table 2. These data also show that when migration stops, the average age of the population increases as observed for Black Caribbean women. Age and sex are the most dominant risk factors for breast cancer, but age also has a large bearing on route to diagnosis [10]and should be considered when examining breast cancer in relation to ethnicity.

In the UK, routine mammographic screening is offered every three years to women aged 50-70 years [8]. A recent review has investigated the reasons for the lower observed uptake of screening among women from ethnic minority backgrounds, but many of the studies included use aggregate ethnic groupings and consider Asian and Black women as homogenous groups which is potentially misleading to communities [26]. The data presented in Figure 1 shows that four out of five Black African and Pakistani women are aged under 50 and as such would not be invited for routine population based mammographic screening. Therefore, initiatives that focus on increasing screening uptake among ethnic minority women would potentially be of little benefit in communities which are largely Pakistani or Black African, whereas initiatives designed to encourage early presentation with symptoms are likely to be of greater relevance. Conversely, the 2021 census data show that one in three women from Black Caribbean backgrounds are now of screening age, and targeted campaigns to ensure awareness in those communities about availability of breast screening programmes would potentially be beneficial.

Lower age standardised breast cancer incidence rates among aggregate ethnic minority groups, South Asians and Blacks, compared to white groups have been previously reported [1]. The common lifestyle risk factors for breast cancer are well described with increased risk associated with increased use of alcohol [27], use of hormone replacement therapy [28] and postmenopausal obesity [29]. Conversely, reduced risk is associated with increased rates of parity and breastfeeding [30]. The differences in breast cancer incidence by ethnicity have been examined in a large prospective cohort study and are largely, if not wholly, explained by differences in the prevalence of known lifestyle and reproductive risk factors, for breast cancer among the different groups [6].

However, the differences in incidence between specific ethnicities within these aggregate groups are not previously described and require further investigation. Large prospective cohort studies can be used to examine variations in breast cancer incidence in these groups taking account of known risk factors. The generation of uniquely reliable estimates of breast cancer incidence, as well as estimates of risk factor prevalence in UK women by ethnicity, age and country of birth is important to predict future trends in incidence among these ethnic groups and future healthcare needs. Variations in the patterns of routes to diagnosis in these ethnic groups will also impact on breast cancer incidence rates and needs to be examined further. Studies have shown screening attendance in asymptomatic women varies by ethnicity and is influenced by acculturation [31, 32], and that health seeking behaviour for cancer symptoms in migrant groups is influenced by low levels of health literacy, adult migration to the UK and fatalistic health beliefs [33–35].

More broadly, there is much interest in addressing health inequalities particularly with respect to ethnicity [11]. Much of the literature describes ethnicity in broad groups which will mask distinct differences between specific ethnic groups and could worsen inequalities by failing to recognise key differences between these groups as we have highlighted here. Where possible, researchers should aim to describe findings in disaggregated ethnic groups where sufficient numbers are available to do so robustly.

The detailed examination of census data presented here contextualised for breast cancer, further highlights the importance of really understanding population demographics in specific ethnic groups for the planning and delivery of healthcare services. Healthcare professionals and leaders working in ethnically dense regions of the country must be cognisant of the current makeup of their local populations so that health care services and public health messages and strategies are nuanced and relevant to those communities where inequalities may be a concern.

Additionally, healthcare service leaders should be considering what future population demographics will be based on current migration patterns so that health services remain relevant to local needs. All services should be designed and developed in discussion with communities to raise awareness about availability and access to them.

## Figures and Tables

**Table 1:** Ethnic groups in the 2001, 2011 and 2021 census in England

Ethnic groups		Year of census	
	2001	2011	2021
**Asian or Asian British**			
Indian	●	●	●
Pakistani	●	●	●
Bangladeshi	●	●	●
Chinese		●	●
Any other Asian background	●	●	●
**Black or Black British**			
Caribbean	●	●	●
African	●	●	●
Any other Black background	●		
Any other Black, African or Caribbean background		●	●
**Mixed**	●	●	●
White and Black Caribbean	●	●	●
White and Black African	●	●	●
White and Asian	●	●	●
Any other Mixed background	●		
Any other Mixed or multiple ethnic background		●	●
**White**			
British	●		
English, Welsh, Scottish, Northern Irish or British		●	●
Irish	●	●	●
Gypsy or Irish Traveller		●	●
Roma			●
Any other White background	●	●	●
**Chinese or other ethnic group**			
Chinese	●		
Any other	●		
**Other ethnic group**			
Arab		●	●
Any other ethnic group		●	●

**Table 2:** Number and median age of Black African, Black Caribbean, Indian, Pakistani and white women in the 2001, 2011 and 2021 national census

Ethnic group			Year of Census			
	2001		2011		2021	
	Number	Median age	Number	Median age	Number	Median age
**Black African**	246 835	27	504 385	28	770 075	31
**Black Caribbean**	301 365	32	316 275	41	335 445	45
**Indian**	517 342	27	685 401	32	922 740	36
**Pakistani**	348 496	22	542 489	25	781 975	28
**White**	22 947 783	37	23 083 076	42	23 353 290	44

**Table 3:** Average age standardised incidence rates for C50 breast cancer by ethnicity in women aged ≥25 years in England during 2011-2019.

Ethnic group	Average annual population (2011 – 2021)	Average annual cases (2011 - 2019)	Adjusted rate per 100,000	(95% CI)
Black African	375 542	282	118.2	(111.6-125.1)
Black Caribbean	243 917	346	146.0	(140.7-151.3)
Indian	560 548	569	134.7	(130.8-138.6)
Pakistani	358 344	304	137.3	(131.5-143.3)
White	17 096 322	35 127	199.6	(198.9-200.3)

## Data Availability

The data presented from the Census in 2001, 2011 and 2021 are publicly available. The cancer registration data in this study are held by the University of Oxford under a data sharing contract with NHS England and are not available for onward data sharing. These data are available by individual application to the Data Access Request Service at NHS England (https://digital.nhs.uk/services/data-access-request-service-dars).

## References

[1] Delon C, Brown KF, Payne NWS, Kotrotsios Y, Vernon Sand, Shelton J (2022). Differences in cancer incidence by broad ethnic group in England, 2013-2017. Br J Cancer.

[2] National Cancer Registration and Analysis Service (2024). Cancer Incidence and Mortality.

[3] Office for National Statistics (2022). Census.

[4] UK Government (2022). List of ethnic groups.

[5] Laux R (2019). 50 years of collecting ethnicity data.

[6] Gathani T, Ali R, Balkwill A, Green J, Reeves G, Beral V (2014). Ethnic differences in breast cancer incidence in England are due to differences in known risk factors for the disease: prospective study. British Journal of Cancer.

[7] National Institute for Health and Care Excellence (2015). Suspected cancer: recognition and referral.

[8] National Health Service Breast Screening Programme When it’s offered - Breast cancer screening.

[9] National Disease Registration Service NE (2024). Routes to Diagnosis.

[10] Gathani T, Cutress R, Horgan K, Kirwan C, Stobart H, Kan SW (2023). Age and sex can predict cancer risk in people referred with breast symptoms. BMJ.

[11] King’s Fund NRaHO (2021). Ethnic health inequalities and the NHS: driving progress in a changing system.

[12] Office for National Statistics (2003). Sex and age by ethnic group.

[13] Office for National Statistics (2020). Ethnic groups by sex and age from the 2011 Census.

[14] Office for National Statistics (2023). Ethnic group by age and sex in England and Wales.

[15] Henson KE, Elliss-Brookes L, Coupland VH, Payne E, Vernon S, Rous B (2020). Data Resource Profile: National Cancer Registration Dataset in England. Int J Epidemiol.

[16] Public Health England (2020). The National Cancer Registration and Analysis Service: a guide to cancer data and working with us.

[17] Office for National Statistics (2013). 2011 Census: Key Statistics for local authorities in England and Wales Table KS201EW: Ethnic group, local authorities in England and Wales.

[18] European Standard Population.

[19] Office for National Statistics (2023). The changing picture of long-term international migration, England and Wales: Census 2021.

[20] legislation.gov.uk British Nationality Act 1948.

[21] Thomas-Hope E (1980). Hopes and Reality in the West Indian Migration to Britain. Oral History.

[22] Domboku T (2018). The Migration History of Balck Africans to Britain. Diasporas and Transnational Entrepreneurship in Global Contexts.

[23] Sturge G (2024). Asylum Statistics_House of Commons Library.

[24] Office for National Statistics (2024). Long-term international migration, provisional: year ending December 2023.

[25] The UK Government Home Office (2024). Why do people come to the UK? To work.

[26] Bolarinwa OA, Holt N (2023). Barriers to breast and cervical cancer screening uptake among Black, Asian, and Minority Ethnic women in the United Kingdom: evidence from a mixed-methods systematic review. BMC Health Serv Res.

[27] Floud S, Hermon C, Simpson RF, Reeves GK (2023). Alcohol consumption and cancer incidence in women: interaction with smoking, body mass index and menopausal hormone therapy. BMC Cancer.

[28] Collaborative Group on Hormonal Factors in Breast Cancer (2019). Type and timing of menopausal hormone therapy and breast cancer risk: individual participant meta-analysis of the worldwide epidemiological evidence. Lancet.

[29] Guo W, Key TJ, Reeves GK (2018). Adiposity and breast cancer risk in postmenopausal women: Results from the UK Biobank prospective cohort. Int J Cancer.

[30] Collaborative Group on Hormonal Factors in Breast Cancer (2002). Breast cancer and breastfeeding: collaborative reanalysis of individual data from 47 epidemiological studies in 30 countries, including 50302 women with breast cancer and 96973 women without the disease. The Lancet.

[31] Anderson de Cuevas RM, Saini P, Roberts D, Beaver K, Chandrashekar M, Jain A (2018). A systematic review of barriers and enablers to South Asian women’s attendance for asymptomatic screening of breast and cervical cancers in emigrant countries. BMJ Open.

[32] Baird J, Yogeswaran G, Oni G, Wilson EE (2021). What can be done to encourage women from Black, Asian and minority ethnic backgrounds to attend breast screening? A qualitative synthesis of barriers and facilitators. Public Health.

[33] Vrinten C, Wardle Jand, Marlow LA (2016). Cancer fear and fatalism among ethnic minority women in the United Kingdom. Br J Cancer.

[34] Waller J, Robb K, Stubbings S, Ramirez A, Macleod U, Austoker J (2009). Awareness of cancer symptoms and anticipated help seeking among ethnic minority groups in England. Br J Cancer.

[35] Williams ED, Whitaker KL, Piano M, Marlow LAV (2019). Ethnic differences in barriers to symptomatic presentation in primary care: A survey of women in England. Psychooncology.

